# Regional differences in severe postpartum hemorrhage: a nationwide comparative study of 1.6 million deliveries

**DOI:** 10.1186/s12884-015-0473-8

**Published:** 2015-02-21

**Authors:** Babette W Prick, Joost F von Schmidt auf Altenstadt, Chantal WPM Hukkelhoven, Gouke J Bonsel, Eric AP Steegers, Ben W Mol, Joke M Schutte, Kitty WM Bloemenkamp, Johannes J Duvekot

**Affiliations:** Department of Obstetrics and Gynecology, Division of Obstetrics & Prenatal Medicine, Erasmus MC, ‘s-Gravendijkwal 230, 3015 CE Rotterdam, The Netherlands; Department of Obstetrics and Gynecology, Maasstad Hospital, Maasstadweg 21, 3079 DZ Rotterdam, the Netherlands; Department of Obstetrics, Leiden University Medical Centre, Albinusdreef 2, 2333 ZA Leiden, The Netherlands; The Netherlands Perinatal Registry, Mercatorlaan 1200, 3528BL Utrecht, The Netherlands; Robinsion Research Institute, School of Pediatrics and Reproductive Health, University of Adelaide, Adelaide, 5000 SA Australia; Department of Obstetrics and Gynecology, Isala Klinieken, Groot Wezenland 20, 8011 JW Zwolle, The Netherlands

**Keywords:** Cohort study, Postpartum hemorrhage, Geographic distribution, Epidemiology, Maternal mortality

## Abstract

**Background:**

The incidence of severe postpartum hemorrhage (PPH) is increasing. Regional variation may be attributed to variation in provision of care, and as such contribute to this increasing incidence. We assessed reasons for regional variation in severe PPH in the Netherlands.

**Methods:**

We used the Netherlands Perinatal Registry and the Dutch Maternal Mortality Committee to study severe PPH incidences (defined as blood loss ≥ 1000 mL) across both regions and neighborhoods of cities among all deliveries between 2000 and 2008. We first calculated crude incidences. We then used logistic multilevel regression analyses, with hospital or midwife practice as second level to explore further reasons for the regional variation.

**Results:**

We analyzed 1599867 deliveries in which the incidence of severe PPH was 4.5%. Crude incidences of severe PPH varied with factor three between regions while between neighborhoods variation was even larger. We could not explain regional variation by maternal characteristics (age, parity, ethnicity, socioeconomic status), pregnancy characteristics (singleton, gestational age, birth weight, pre-eclampsia, perinatal death), medical interventions (induction of labor, mode of delivery, perineal laceration, placental removal) and health care setting.

**Conclusions:**

In a nationwide study in The Netherlands, we observed wide practice variation in PPH. This variation could not be explained by maternal characteristics, pregnancy characteristics, medical interventions or health care setting. Regional variation is either unavoidable or subsequent to regional variation of a yet unregistered variable.

**Electronic supplementary material:**

The online version of this article (doi:10.1186/s12884-015-0473-8) contains supplementary material, which is available to authorized users.

## Background

Severe postpartum hemorrhage (PPH) is a major obstetric complication and an important cause of maternal morbidity and mortality worldwide [1-4]. It is reported to occur in 2-6% of pregnancies [3,5] and its incidence has increased over the last decade [6-13]. The World Health Organization defines severe PPH as blood loss of ≥ 1000 mL [14], independent of the mode of delivery, i.e. vaginal delivery or Cesarean Section (CS).

Carolli reported variation in severe PPH incidence worldwide varying from 0.3 to 3.8% in Africa, 0.7 to 2.7% in Asia and 1.7 to 5.5% in Europe [5]. Since the incidence of severe PPH in several developed countries is increasing, its assessment at the national and regional levels is important. Intercountry variation, particularly between developed and developing countries, may result from differences in medical interventions or health care systems. At a national level, regional differences are also observed, for example in California, where 3-fold differences among regions have been reported [15]. Exploring reasons for these differences is crucial before improvements in clinical practice can be established. Differences in populations may be an explanation for the variation in severe PPH between countries and regions. For this reason, adjustments for the characteristics of mothers and pregnancies seem necessary for a better understanding of the variation.

In this paper, we studied the incidence of severe PPH in the 12 provinces and four largest cities in the Netherlands, as well as in the neighborhoods of the two largest cities. We hypothesized that variation may be due to the combined effect of maternal and pregnancy characteristics, medical interventions and variation in PPH policy in hospital and community midwife practices. As PPH-related mortality accounts for almost 8% of all direct maternal deaths in the period 1993–2005 [16], we also studied the severe PPH-related mortality per region.

## Methods

We studied all deliveries registered in the Netherlands Perinatal Registry (PRN) in the period 2000–2008. Deliveries with a gestational age below 24^+0^ weeks were excluded. We studied the period 2000–2008 as during collection and analyses the data regarding more recent years were not available. The PRN is a linked national registry in which information on 96% of all pregnancies and pregnancy outcomes are registered by care providers [17]. Predefined information is directly extracted from the mothers’ record and sent to the PRN. The PRN registers both data from community midwife practices (LVR1, primary care) providing care to women with low-risk pregnancies and data from hospitals providing care to women with an increased perinatal risk by obstetricians (LVR2, secondary/ tertiary care). Data on active third stage management and professional performance are not available. Neonatal admissions and complications are registered by pediatricians (LNR) and are also incorporated in this registry [17]. Data presented in this study were anonymized: they cannot be related to individual women.

This study was approved by the privacy committee of the PRN. Ethical approval is not needed for this type of study in the Netherlands.

The Netherlands comprises 12 provinces. The eight tertiary care hospitals are located in six of the provinces (Noord-Holland, Zuid-Holland, Utrecht, Gelderland, Limburg and Groningen) while teaching hospitals are present in all provinces. In three of the four largest cities (Amsterdam, Rotterdam and Utrecht) a tertiary care hospital is located while The Hague, the fourth largest city, lacks such a facility. In the Netherlands women with low-risk pregnancies deliver under responsibility of independent community midwife practices or general practitioners. Of these low-risk women 76% deliver at the hospital and 24% deliver at home (Table 1) [18]. Women with high-risk pregnancies are cared for by gynecologists and deliver in hospital.

**Table 1:** **Maternal characteristics, pregnancy characteristics, medical interventions, health care setting and severe PPH incidences**

	**All**		**Spontaneous**		**Assisted vaginal delivery**		**Elective CS**		**Emergency CS**	
	**%**	**PPH incidence**	**%**	**PPH incidence**	**%**	**PPH incidence**	**%**	**PPH incidence**	**%**	**PPH incidence**
*Maternal characteristics*										
Age, n (%)										
≤ 35 years	85.6	4.4	86.0	4.3	88.4	6.2	78.9	3.9	83.6	3.0
> 35 years	14.4	5.1	14.0	4.8	11.6	7.7	21.1	5.5	16.4	4.3
Parity										
0	46.2	5.0	39.1	5.1	82.1	6.6	42.0	3.8	67.7	2.8
≥ 1	53.8	4.0	60.9	3.9	17.9	5.5	58.0	4.6	32.3	3.9
Ethnicity										
Western	84.5	4.6	83.9	4.4	88.2	6.6	86.7	4.1	83.5	3.2
Non-Western	15.5	4.1	16.1	4.0	11.8	4.8	13.3	5.3	16.5	3.3
Social economic status										
Highest (> p 80)	17.2	4.9	17.0	4.7	18.2	7.2	18.0	4.7	17.0	3.3
Moderate (p20-80)	57.8	4.5	57.8	4.3	58.4	6.4	58.9	4.1	57.0	3.2
Lowest (< p20)	25.0	4.2	25.3	4.2	23.5	5.7	23.1	4.2	26.0	3.0
*Pregnancy characteristics*										
Singleton pregnancy	98.0	4.3	98.6	4.2	98.2	6.2	94.1	3.5	96.1	3.0
Multiple pregnancy	2.0	12.1	1.4	11.1	1.8	17.4	5.9	16.0	3.9	7.5
Gestational age										
≥ 37 weeks	93.3	4.4	94.3	4.2	95.2	6.3	81.9	3.7	91.0	3.1
< 37 weeks	6.7	6.0	5.7	5.9	4.8	8.3	18.1	6.6	9.1	3.9
Pre-eclampsia										
Yes	2.2	7.4	1.3	8.9	2.7	12.1	8.1	4.6	4.8	4.1
No	97.8	4.2	98.7	4.1	97.3	6.1	91.9	3.9	95.2	2.8
Birth weight neonate										
< 10^th^ percentile	9.7	3.4	9.5	3.5	8.5	4.4	12.0	3.0	11.5	1.7
10-90^st^ percentile	79.7	4.3	81.0	4.2	79.0	5.9	75.6	4.3	72.3	3.0
> 90^st^ percentile	10.5	7.0	9.5	6.8	12.5	10.7	12.4	5.4	16.2	5.0
Perinatal death										
Yes	0.8	7.5	0.8	7.3	0.4	10.3	1.0	8.8	0.7	5.5
No	99.2	4.5	99.2	4.3	99.6	6.4	99.0	4.2	99.3	3.2
*Medical interventions*										
Induction of labor										
Yes	14.8	6.2	14.0	6.3	18.7	8.5	NA		29.5	3.6
No	85.2	4.2	86.0	4.0	81.3	5.9	NA		70.5	3.0
Delivery										
Spontaneous	74.7	4.3	NA		NA		NA		NA	
Assisted vaginal delivery	10.7	6.4	NA		NA		NA		NA	
Elective CS	6.5	4.3	NA		NA		NA		NA	
Emergency CS	8.1	3.2	NA		NA		NA		NA	
Perineal laceration										
Intact/ ≤ 1^st^ degree rupture	71.4	3.8	74.0	3.7	14.8	5.1	NA		99.2	3.2
≥ 2^nd^ degree rupture/ episiotomy	28.6	6.3	26.0	6.2	85.2	6.6	NA		0.8	3.6
Manual placenta removal										
Yes	1.9	58.3	2.0	59.6	3.8	53.2	NA		NA	
No	98.1	3.5	98.0	3.2	96.2	4.6	NA		NA	
*Health care setting*										
Delivery										
Home (community midwife)	24.2	2.3	32.4	2.2	NA		NA		NA	
Hospital (community midwife)	10.9	4.2	14.6	4.2	NA		NA		NA	
Tertiary care Hospital	5.8	7.4	4.8	7.3	7.3	8.1	11.5	7.9	8.7	6.5
Teaching Hospital	30.4	5.6	25.1	6.0	46.9	6.6	44.4	4.0	45.4	3.1
Non-teaching Hospital	28.8	4.7	23.2	5.0	45.9	5.9	44.1	3.6	45.9	2.7

Total deliveries: 1599867, PPH unknown: 44859, Mode of delivery unknown: 2350.

NA = not applicable, CS = cesarean.

The incidence of severe PPH was analyzed per province and in the four largest cities because previous studies reported that perinatal outcomes in these cities are inferior to those in the other regions (provinces) of the Netherlands [19-21]. For the two largest cities, Amsterdam and Rotterdam, the incidence of severe PPH was analyzed across neighborhoods. Women were classified as part of every province, city and neighborhood using the four-digit zip codes of the women’s address as geographical classifier. Therefore, a woman from a rural area, delivering in a tertiary hospital, was analyzed as woman from that rural region. Maternal mortality numbers were independently obtained from the Maternal Mortality Committee of the Dutch Society of Obstetrics and Gynecology [16].

### Statistical analysis

We studied crude incidence of severe PPH across Dutch provinces, in the four largest cities, and across neighborhoods in the two largest cities (Amsterdam and Rotterdam). PPH-related maternal mortality across regions was studied in a similar way. Crude severe PPH incidences were tabulated, as well as crude incidences after exclusion of multiple pregnancies. Data were stratified for mode of delivery as PPH incidence varies according to mode of delivery [22].

The distribution of the severe PPH incidences was projected on a map of the Netherlands. For this figure, quartiles of incidences were chosen as cutoffs. We performed logistic multilevel regression analyses to explore the origin of the observed geographical variation using hospitals and community midwife practices as the second level in these analyses. For this purpose, each hospital and community midwife practice was labeled using an anonymized code.

We performed six model specifications following the same pattern and all models were fit on the exact same data. First, we estimated a model including regions only to assess the outcome (incidence of severe PPH) across regions. Then, we repeated the analysis after addition of covariates, which were added block-wise. Every subsequent model thus consisted of the first model and one specific block of covariates. The second model included maternal characteristics (age, parity, ethnicity, socioeconomic status [SES]) as covariates, the third model pregnancy characteristics (singleton, gestational age, birth weight, pre-eclampsia, perinatal death), the fourth model medical interventions (induction of labor, mode of delivery, perineal laceration, placental removal) and the fifth model health care setting (place of delivery). Finally, the last model was estimated after inclusion of all blocks (maternal characteristics, pregnancy characteristics, medical interventions and health care setting).

We used the following definitions. Severe PPH was defined as peripartum blood loss ≥ 1000 mL in the 24 hours following delivery. We categorized maternal age in ≤ 35 and > 35 years and parity into nulliparous women (i.e., women who had never given birth) or multiparous women (i.e., women who had given birth at least once). Ethnicity was categorized in Western or non-Western. Social economic status (SES) was derived from the recorded zip code of the women [23]. Gestational age at delivery was categorized into ≥ 37 weeks and < 37 weeks. Pre-eclampsia was defined as a diastolic blood pressure of minimal 90 mmHg in the presence of proteinuria after 20 weeks of gestation [24]. Birth weight percentiles were derived from sex and parity specific growth curves [25] and considered to be undefined for multiple pregnancies and neonates with congenital anomalies or perinatal death. We distinguished between spontaneous onset of labor and induction of labor. Mode of delivery was categorized into spontaneous vaginal, assisted vaginal delivery, elective CS or emergency CS. Perineal laceration was split into ‘none or first degree’ or ‘at least second degree’ and a distinction between spontaneous and manual placental delivery was made. The Dutch health care setting was described in more detail above. In case of a discrepancy between LVR1 and LVR2 source data, LVR2 data prevailed, with the exception for the variable ethnicity.

A funnel plot was created using Excel. Tests for differences between groups were performed using SPSS; multilevel analyses were performed using proc glimmix in SAS version 9.3 software.

## Results

We studied a total of 1599867 women. General characteristics of their deliveries are tabulated in Table 1. Severe PPH was reported in 69719 (4.5%) of all deliveries; and severe PPH incidence increased from 3.8 to 5.8% during the study period (p < 0.001). PPH was unknown in 44859 deliveries.

When classified according to mode of delivery, the incidence of severe PPH was 4.3% after spontaneous delivery, 6.4% after assisted vaginal delivery and 4.3% and 3.2% after elective CS and emergency CS respectively.

### Severe PPH incidence per region

Additional file 1 tabulates the crude incidence across regions.

#### Crude incidence

**Figure 1:**
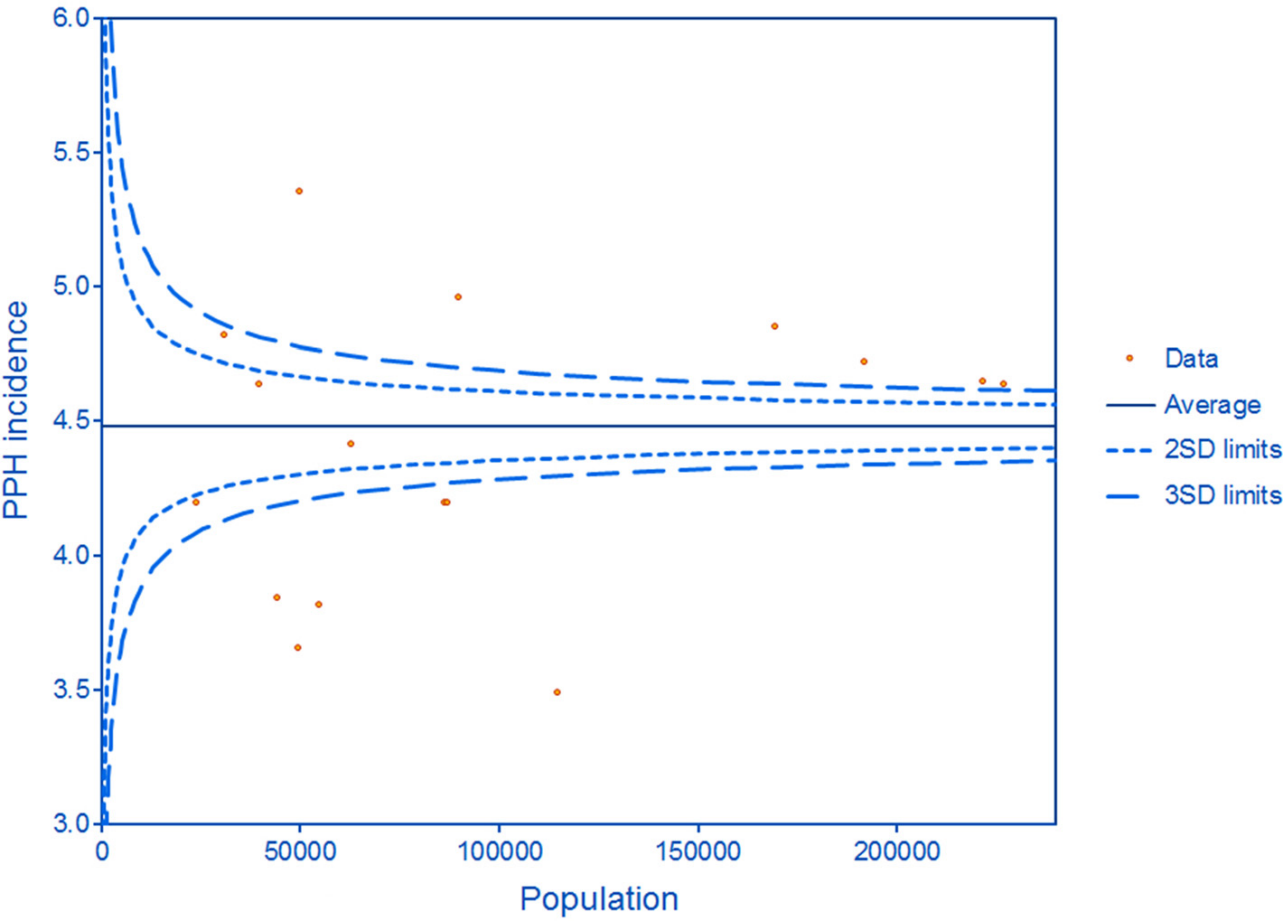
**Funnel plot: variation in regional severe PPH incidence related to regional population size.**

**Figure 2:**
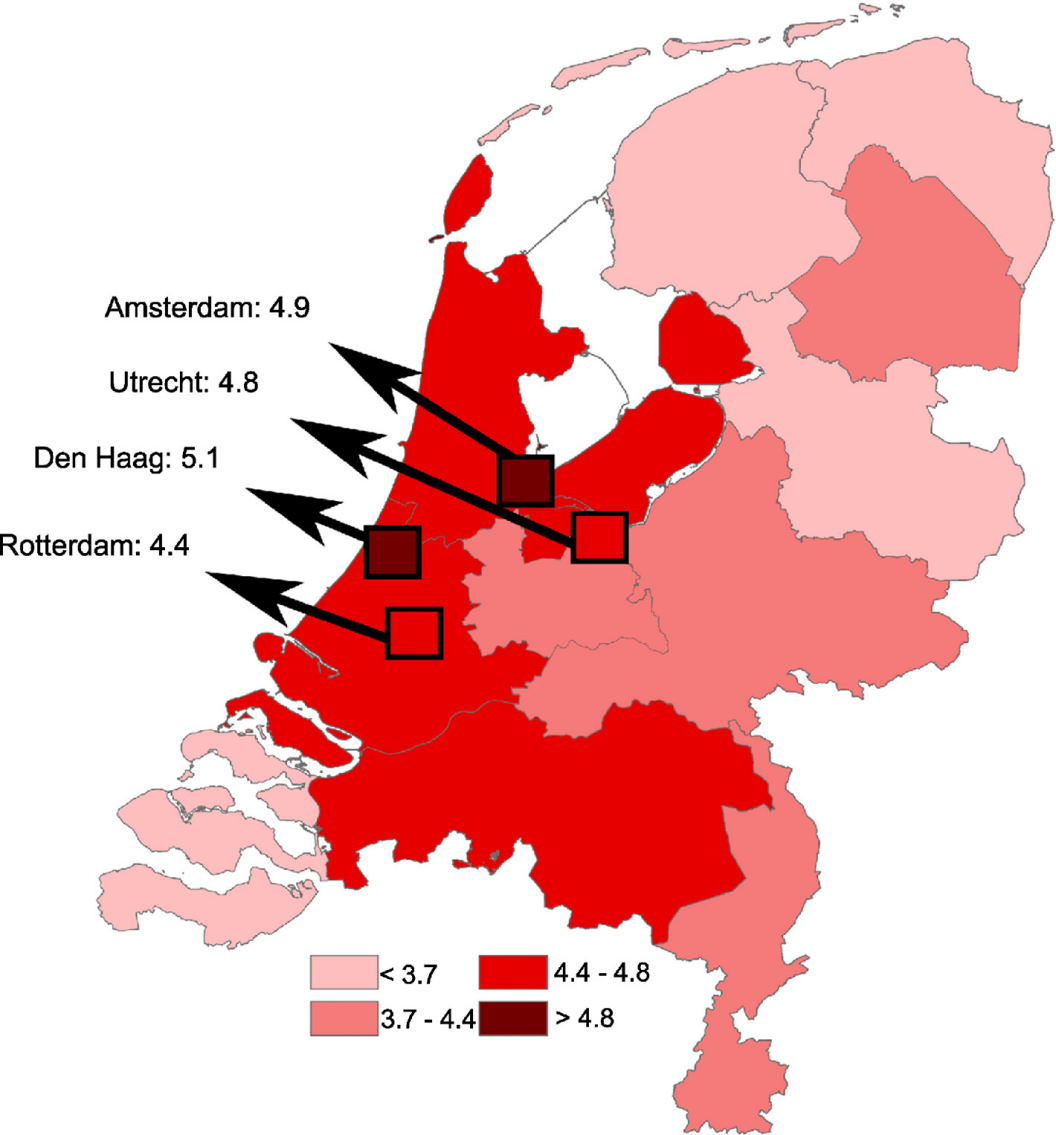
**Crude incidence of severe PPH following spontaneous delivery per region.**

Figure 1 demonstrates the wide variation in crude incidence per region in relation to the regional population size. After stratification by mode of delivery, the incidence of severe PPH was up to 3 times higher in the region with the highest incidence compared to that with the lowest. In Figure 2 the crude incidence of PPH following spontaneous delivery is demonstrated per region. After spontaneous delivery, the incidence ranged from 3.3% to 5.1%. After assisted vaginal delivery this range was 4.8% to 7.9% while the ranges after elective and emergency CS were 2.7% to 6.8% and 1.5% to 4.9%, respectively. Without stratification for mode of delivery, the crude severe PPH incidence in the four cities was 4.9% compared to 4.4% in the provinces (p < 0.001).

#### Singleton pregnancies

After exclusion of multiple pregnancies, the average crude incidence of severe PPH was 4.2% after spontaneous delivery, 6.2% after assisted vaginal delivery, and 3.5% and 3.0% after elective and emergency CS, respectively (Additional file 1). In most regions, the incidence of severe PPH decreased after exclusion of multiple pregnancies. When classified per mode of delivery, regional ranks in the incidence of severe PPH remained similar.

#### Logistic multilevel regression analyses

Table 2 shows the OR’s of severe PPH per region taking into account the level ‘hospital or community midwife practice’. Crude, unadjusted regional OR’s of the first model ranged from 0.80 to 1.10. The OR’s of severe PPH in the four largest cities were similar to those in the provinces.

**Table 2:** **Results of logistic multilevel regression analyses of PPH per region**

	**Unadjusted**	**Adjusted for**					
		**Maternal characteristics**	**Pregnancy characteristics**	**Medical interventions**	**Health care setting**	**All**	**% Change in OR: unadjusted vs adjusted for all**
*Region*							
Amsterdam^a^	1.02 (0.92-1.13)	1.12 (1.01-1.24)	1.08 (0.96-1.20)	1.02 (0.92-1.13)	1.00 (0.91-1.10)	1.05 (0.94-1.17)	3
Rotterdam^a^	0.94 (0.85-1.04)	1.05 (0.94-1.17)	0.97 (0.86-1.08)	0.91 (0.82-1.02)	0.86 (0.78-0.94)	0.90 (0.80-1.01)	−5
The Hague^a^	1.00 (0.89-1.12)	1.04 (0.93-1.16)	0.99 (0.88-1.12)	0.91 (0.81-1.03)	0.91 (0.82-1.02)	0.84 (0.74-0.94)	−20
Utrecht^a^	0.89 (0.82-0.97)	0.92 (0.85-1.00)	0.94 (0.86-1.02)	1.00 (0.91-1.10)	0.89 (0.82-0.96)	1.02 (0.92-1.13)	12
Groningen	0.80 ( 0.69-0.92)	0.83 (0.72-0.96)	0.77 (0.66-0.90)	0.73 (0.63-0.84)	0.75 (0.66-0.86)	0.71 (0.61-0.82)	−13
Friesland	0.88 (0.76-1.03)	0.91 (0.79-1.06)	0.78 (0.66-0.91)	0.73 (0.63-0.85)	0.86 (0.75-0.98)	0.70 (0.60-0.82)	−26
Drente	0.83 (0.72-0.95)	0.85 (0.74-0.97)	0.78 (0.67-0.90)	0.73 (0.63-0.83)	0.80 (0.70-0.90)	0.71 (0.62-0.82)	−16
Overijssel	0.91 (0.82-1.01)	0.92 (0.93-1.03)	0.84 (0.74-0.94)	0.81 (0.73-0.91)	0.92 (0.83-1.01)	0.83 (0.74-0.93)	−10
Gelderland	1.01 (0.93-1.08)	1.02 (0.94-1.09)	0.95 (0.88-1.03)	0.92 (0.85-1.00)	1.02 (0.95-1.09)	0.92 (0.85-1.01)	−9
Utrecht	Ref	Ref	Ref	Ref	Ref	Ref	
Noord Holland	1.10 (1.01-1.21)	1.12 (1.02-1.22)	1.06 (0.97-1.17)	1.02 (0.93-1.12)	1.08 (1.00-1.18)	0.98 (0.89-1.08)	−13
Zuid Holland	1.05 (0.96-1.15)	1.07 (0.98-1.16)	1.03 (0.94-1.14)	0.96 (0.88-1.05)	0.99 (0.91-1.07)	0.89 (0.81-0.98)	−18
Zeeland	1.05 (0.87-1.26)	1.07 (0.89-1.28)	0.96 (0.79-1.16)	0.98 (0.81-1.18)	0.94 (0.80-1.12)	0.88 (0.73-1.07)	−19
Noord Brabant	1.03 (0.94-1.13)	1.04 (0.95-1.14)	1.02 (0.92-1.12)	0.95 (0.86-1.04)	1.02 (0.94-1.11)	0.97 (0.88-1.06)	−7
Limburg	1.04 (0.92-1.17)	1.05 (0.93-1.18)	1.00 (0.88-1.14)	0.90 (0.80-1.02)	0.97 (0.87-1.08)	0.86 (0.76-0.98)	−20
Flevoland	1.07 (0.94-1.23)	1.10 (0.96-1.26)	0.93 (0.80-1.08)	0.93 (0.81-1.07)	1.05 (0.93-1.20)	0.87 (0.74-1.01)	−24

^a^City.

Level used was the unique code for each hospital and community midwife practice.

Maternal characteristics: Age, Parity, Ethnicity, SES.

Pregnancy characteristics: Singleton pregnancy, Gestational age, Birth weight, Pre-eclampsia, Perinatal Death.

Medical interventions: Induction of labor, Mode of delivery, Perineal laceration, Placental removal.

Health care setting: Place of delivery.

Adjustment for maternal characteristics led to higher OR’s compared to the first unadjusted model. Most differences were relatively small except for the cities Amsterdam and Rotterdam. Compared to the first unadjusted model, adjustment for pregnancy characteristics revealed overall lower OR’s while adjustment for medical interventions led to an ambiguous effect on the OR’s. Adjustment for health care setting also produced an ambiguous effect with mainly higher OR’s compared to the first unadjusted model. Of the four blocks of covariates that were added to the first unadjusted model, pregnancy characteristics and health care had the strongest impact on the OR’s.

Compared to the unadjusted model, adjustment for all blocks simultaneously had the largest effect on the severe PPH OR’s, although this effect was ambiguous.

After adjustment for all blocks, the regional variation in severe PPH OR was largest.

After stratification for mode of delivery, adjustment for all blocks demonstrated ambiguous effects for each mode of delivery too (data not shown).

### Severe PPH incidence in Amsterdam

Additional file 2 tabulates the crude incidence of PPH across neighborhoods in Amsterdam.

#### Crude incidence

The average crude PPH incidence was 5.0%. There was a wide variation in incidence across neighborhoods with differences between neighborhoods up to 7.3%, depending on the mode of delivery. After spontaneous delivery, the incidence ranged from 3.6% to 6.4%, after assisted vaginal delivery from 3.8% to 10.4% while the ranges after elective and emergency CS showed the widest variation: 1.6% to 8.9% and 0.8% to 5.3%, respectively.

#### Singleton pregnancies

After exclusion of multiple pregnancies, the average crude incidence was 4.9% after spontaneous delivery, 7.4% after assisted vaginal delivery, 3.7% after elective CS and 2.4% after emergency CS.

### Severe PPH incidence in Rotterdam

Additional file 3 tabulates the crude incidence of PPH across neighborhoods in Rotterdam.

#### Crude incidence

The average crude PPH incidence was 4.4%. Again, differences between neighborhoods were large, with a maximum difference of 8.0%. The incidence differed from 3.8% to 6.2% for spontaneous delivery, from 4.1% to 12.1% for assisted vaginal delivery while variation was largest for elective and emergency CS: 1.8% to 6.6% and 0% to 4.9%, respectively.

#### Singleton pregnancies

Average crude incidence after exclusion of multiple pregnancies was 4.3% after spontaneous delivery, 5.3% after assisted vaginal delivery and 3.4 and 3.2% after elective and emergency CS.

### PPH-related mortality

Almost eight percent of total Dutch maternal mortality was related to PPH (see Table 3). PPH-related mortality varied from 0.16% to 0.59% across regions. It showed a pattern dissimilar from the pattern of crude severe PPH incidence across regions.

**Table 3:** **Crude maternal mortality per region**

	**Deliveries** ^**a**^	**Severe PPH** ^**a**^	**Total mortality (all causes)** ^**b**^	**Mortality, due to severe PPH** ^**b**^ **(% of total mortality)**
	**n**	**n**	**n**	**n**
*Region*				
Amsterdam^c^	90738	4656	9	0
Rotterdam^c^	63571	2891	17	1
Den Haag^c^	53509	2812	9	0
Utrecht^c^	37098	1583	4	0
Groningen	50823	1912	5	0
Friesland	56079	2201	7	1
Drente	44942	1795	6	1
Overijssel	118003	4227	15	2
Gelderland	197320	9575	15	1
Utrecht	93408	3828	19	3
Noord Holland	173166	8613	16	0
Zuid Holland	226040	10843	20	2
Zeeland	27505	1047	6	1
Noord Brabant	230043	11081	20	1
Limburg	88794	3833	10	0
Flevoland	40107	1941	5	0
Unknown region	8721	430	4	1
*Total*	1599867	73268	187	14 (7.5%)

^a^Data derived from PRN.

^b^Data derived from Maternal Mortality Committee.

^c^City.

## Discussion

In this study, we found a wide variation in severe PPH incidence across regions (provinces, cities) and neighborhoods in the Netherlands, with crude incidences ranging from below 2% to well over 8%. In women with elective and emergency CS, the variation in severe PPH incidence was the most extreme. As expected, exclusion of multiple pregnancies decreased crude incidences. The incidence of severe PPH was higher in the four cities. After logistic multilevel regression analyses, in which we controlled for maternal characteristics, pregnancy characteristics, medical interventions and health care setting, the variation per region even increased (Table 2). Apparently, the epidemiological data cannot elucidate the sources of the remnant variation: results imply they are random or subsequent to regional variation of a yet unregistered variable. Results from our study imply that this unregistered variable is most likely to be found among differences in care and management (professional performance). It is known that adherence to clinical guidelines in on average questionable [26,27] and differences may exist between local protocols. Therefore, results of this study should lead to audit programs to investigate causes.

Regional differences in PPH incidence have been described previously. Carolli et al. [5] reported similar variation in severe PPH incidence worldwide while Fong et al. [15] reported variation in California. These studies did not explore the causes of these variations. In addition, Lu et al. found large differences across hospitals in California and also found higher incidences of obstetrical trauma, chorioamnionitis, and protracted labor (used as proxy of the quality of health care) in hospitals with a high PPH incidence [28].

Known risk factors for PPH can be divided in maternal characteristics, pregnancy characteristics, medical interventions and health care setting. Higher PPH incidences have been reported in women with increased age or decreasing SES, while data regarding ethnicity and parity are contradictive [3,29-33]. Beside these maternal characteristics, other risk factors for PPH that have been described in the literature are induction of labor, augmentation of labor, perineal laceration, manual placental removal and an increasing fetal weight [3,29,30]. Additionally, variation in Dutch obstetric care, regarding the obstetric intervention rate, has previously been described [34]. Although these risk factors varied to a large extent across regions (data not shown), PPH variation remained after adjustment for these factors.

In this study, we presented the geographical distribution while taking into account hospital or community midwife practice (because on average 24% of women in the Netherlands deliver at home [18]). Regarding place of birth, no relation to the occurrence of PPH was reported by Davis et al. in a recent study [35] while de Jonge et al. reported lower rates of PPH in women with planned home birth [36].

The incidence of severe PPH depended on mode of delivery, and classified by mode of delivery, variation was not uniform. Although this finding has been reported in previous studies, too [5,9,11,37], the relatively low severe PPH incidence after (emergency) CS was remarkable. Overall, mean blood loss during delivery is higher in case of CS [38]. An explanation for the lower rate of severe PPH might be that during surgery adequate measures to control the bleeding are more quickly available than at the delivery ward. Alternatively, there might be the possibility of registration errors. The magnitude of such errors, however, is not expected to vary across regions and will therefore not have influenced the variation across regions to a great extent.

In this study, almost 8% of maternal mortality was related to PPH; the incidence of maternal mortality related to PPH varied across regions between 0.16 and 0.59%. In California, PPH-related mortality across regions has been described to vary between 0.08 and 0.21% [15]. In our study, variation was thus larger. An explanation for the wider variation may be that our dataset was smaller. Haeri et al. reported 13% of maternal mortality in developed countries to be caused by PPH [39]. The variation of mortality in our study was not comparable to the variation pattern of severe PPH, unlike findings of Fong et al. [15]. Again, mortality numbers in our study were very small.

The following limitations of this study have to be considered. Despite our large dataset, the number of deliveries in some neighborhoods were relatively small. Also, a small number of care providers did not participate in the PRN (5% of community midwife practices, 1% of gynecologists), but it is that unlikely non-participation is related to the occurrence of PPH. Another limitation concerns data on other PPH determinants. Data on body mass index (BMI) data were unavailable, which is worrisome as BMI is proposed to be a risk factor for PPH [40-42] and Dutch public health reports show regional BMI variation [43]. However, at the aggregate level epidemiological patterns apparently do not relate. Also, data on active third stage management and professional performance were unavailable.

Although literature has shown that estimation of the amount of blood loss is often inaccurate [38,44,45], blood loss is not routinely weighed in the Netherlands in all deliveries. After vaginal deliveries blood loss is mostly estimated in case of normal blood loss and usually weighed in case of persisting blood loss. In case of CS, abdominal blood loss is collected through suction in a measuring pot and estimated postoperatively based on the amount of fluid in the pot and the vaginal blood loss. To our knowledge, no validation studies on blood loss estimation have been performed in the Netherlands. Blood loss is recorded in the PRN dataset as a binary variable with a cutoff of 1000 mL (irrespective of the mode of delivery). This cutoff was derived from the definition of severe postpartum hemorrhage by the World Health Organization [14] and is the most widely used in literature. While blood loss of 1000–2000 mL is usually not a major threat to the maternal condition in developed countries, we believe this cutoff is accurate. Results with this cutoff are more trustworthy due to the (relatively) large number of cases than with a higher cutoff. However, in our dataset the method for blood loss measurement is not registered and might vary. Consequently, reported differences in severe PPH incidence might have their origin also in regional differences in methods of measurements. Despite the inaccuracy of the blood estimation procedure in general [46], we assume the cutoff of 1000 mL to be sufficiently accurate for our purposes, as the great majority of such cases will involve weighed blood loss. Bias through differences in these estimations is expected to be small as, if present, misclassification is expected to be similar across regions. The major weakness in the dataset is the lack of registration of preventive and therapeutic measures.

The major strength of this study is the very large dataset containing 96% of all deliveries in the Netherlands in the period 2000–2008. These numbers strengthen external validity of the study. Since data were extracted electronically from the medical records, data are trustworthy. Data were prechecked through built-in checks and through post-hoc algorithms of the PRN; note however that the dataset was anonymized excluding the potential for individual retrospective checks.

## Conclusions

A large variation in severe PPH incidence exists nationwide in the Netherlands. This variation could not be explained by maternal characteristics, pregnancy characteristics, medical interventions or health care setting. Regional variation may be random or subsequent to regional variation of a yet unregistered variable.

## Additional files

Additional file 1:
**Crude incidences of PPH per region.**
Additional file 2:
**Crude incidences of PPH in Amsterdam.**
Additional file 3:
**Crude incidences of PPH in Rotterdam.**

## Abbreviations

BMI: Body Mass Index
CS: Cesarean Section
MMC: Maternal Mortality Committee
PRN: Netherlands Perinatal Registry
PPH: Postpartum Hemorrhage
SES: Social Economic Status
