# Supplementary material for: Regional differences in severe postpartum hemorrhage: a nationwide comparative study of 1.6 million deliveries
Source: BMC Pregnancy Childbirth. 2015 Feb 21;15:43. doi: 10.1186/s12884-015-0473-8 (PMC4341225; doi:10.1186/s12884-015-0473-8)
Supplement: Additional file 1: — Crude incidences of PPH per region. [file 12884_2015_473_MOESM1_ESM.pdf]

Additional file

Additional file 1 Crude incidences of PPH per region

	Total	Spontaneous delivery		Assisted vaginal delivery		Elective CS		Emergency CS	
	All	All	Singleton pregnancies	All	Singleton pregnancies	All	Singleton pregnancies	All	Singleton pregnancies
Region									
Amsterdam[a]	5.0	4.9	4.9	7.6	7.4	4.9	3.8	2.6	2.4
Rotterdam[a]	4.4	4.4	4.3	5.5	5.3	4.3	3.4	3.3	3.2
The Hague[a]	5.4	5.1	5.0	7.6	7.3	6.4	5.5	4.2	4.0
Utrecht[a]	4.8	4.8	4.6	5.9	5.7	4.8	4.1	3.1	2.6
Groningen	3.7	3.4	3.3	4.8	4.5	3.7	3.3	3.0	2.9
Friesland	3.8	3.6	3.4	6.0	5.8	2.9	2.4	3.1	3.1
Drente	3.8	3.8	3.7	6.3	6.1	2.9	2.4	1.5	1.4
Overijssel	3.5	3.3	3.2	5.4	5.2	3.1	2.4	2.6	2.5
Gelderland	4.7	4.3	4.2	6.7	6.4	6.0	5.2	4.9	4.6

Utrecht	4.2	4.1	4.0	5.7	5.4	4.3	3.7	2.6	2.1
Noord									
Holland	4.9	4.8	4.7	7.9	7.7	3.4	2.7	2.1	2.0
Zuid Holland	4.6	4.5	4.4	6.3	6.1	4.5	3.6	3.8	3.6
Zeeland	4.2	3.5	3.4	6.8	6.6	6.8	6.2	4.5	4.5
Noord									
Brabant	4.6	4.5	4.4	6.3	6.1	4.4	3.8	3.6	3.3
Limburg	4.2	4.3	4.2	5.9	5.7	2.7	2.2	2.0	1.8
Flevoland	4.6	4.7	4.6	5.2	5.1	4.5	3.7	2.4	2.2
Total	4.5	4.3	4.2	6.4	6.2	4.3	3.5	3.2	3.0

[a] City

CS = cesarean section
