# Supplementary material for: Regional differences in severe postpartum hemorrhage: a nationwide comparative study of 1.6 million deliveries
Source: BMC Pregnancy Childbirth. 2015 Feb 21;15:43. doi: 10.1186/s12884-015-0473-8 (PMC4341225; doi:10.1186/s12884-015-0473-8)
Supplement: Additional file 3: — Crude incidences of PPH in Rotterdam. [file 12884_2015_473_MOESM3_ESM.pdf]

Additional file 3 Crude incidences of PPH in Rotterdam

	Total	Spontaneous		Assisted vaginal		Elective CS		Emergency CS	
	All	All	Singleton pregnancies	All	Singleton pregnancies	All	Singleton pregnancies	All	Singleton pregnancies
Neighborhood									
Stadscentrum	4.5	4.2	4.2	6.0	6.1	5.7	4.5	3.8	3.9
Charlois	4.2	4.0	3.8	5.3	5.0	3.8	2.8	4.5	4.4
Delfshaven	4.1	4.0	3.9	4.1	4.2	4.4	3.7	4.9	5.0
Feijenoord	3.7	3.8	3.8	4.2	4.2	3.3	3.0	2.1	2.0
Hilligersberg									
Schiebroek	5.8	6.2	6.2	7.1	7.1	3.8	3.6	1.4	1.4
Hoogvliet	5.0	5.0	4.9	5.8	4.9	4.6	3.9	3.6	3.3
Hoek van Holland	5.0	4.4	4.5	12.1	9.5	3.1	3.3	3.9	4.0
Ijsselmonde	4.2	4.5	4.3	4.5	4.6	4.0	2.8	1.2	1.0
Kralingen Crooswijk	4.4	4.4	4.3	5.8	5.6	4.4	3.4	2.9	2.7
Noord	4.4	4.4	4.3	6.5	6.0	1.8	1.9	2.3	2.4

Overschie	5.1	5.6	5.5	5.2	4.7	3.3	3.5	0.8	0.9
Pr Alexander	4.8	4.3	4.3	6.1	6.0	6.6	4.7	4.9	4.7
Pernis	5.0	5.1	4.8	8.2	8.5	4.6	0	0	0
Total	4.4	4.4	4.3	5.5	5.3	4.3	3.4	3.3	3.2

CS = cesarean section
